# Age-friendly initiatives – GHANA

**DOI:** 10.1016/j.jnha.2024.100246

**Published:** 2024-04-25

**Authors:** Sally Yalley, Akye Essuman, Patrick Adjei, Brian Lawlor, Roman Romero-Ortuno

**Affiliations:** a Global Brain Health Institute, Trinity College Dublin, Dublin, Ireland; b Alzheimer’s and Related Disorders Association of Ghana, Tema, Ghana; c University of Health and Allied Sciences, Ho, Ghana; d Ghana College of Physicians and Surgeons, Accra, Ghana; e University of Ghana Medical School, Accra, Ghana; f Korle Bu Teaching Hospital, Accra, Ghana

**Keywords:** Age-friendly, Initiatives, Ghana, Africa, Dementia

## Abstract

The global increase in the population of older persons has profound inter-sectoral implications, necessitating the development of age-friendly initiatives at the global and national levels. While progress has been relatively slower across Sub-Saharan African countries, highlighting existing commendable initiatives is essential to identify the current gaps and promote the development of strategies and interventions to promote age-friendly societies. This mini-review highlights some of the key initiatives in Ghana in the areas of policy, healthcare, finance, social services, education and research and in promoting dementia-friendly communities.

## 1 Ageing in GHANA

In the face of global population ageing, Sub-Saharan African nations, including Ghana, are projected to experience the highest increase in older people, by up to 230% by 2050 [1]. In the last six decades, the population of people aged 60 years and older in Ghana has increased by about tenfold from about 200,000 in 1960 to over 2 million in 2021 [2]. This rapid demographic shift poses a pressing challenge to implement timely policies and interventions, ensuring that health and social care systems are equipped to address the evolving needs of an ageing population.

## 2 Ageing policies

The United Nations (UN) has declared a decade of Healthy Ageing from 2021 to 2030, with healthy ageing described as “developing and maintaining the functional ability that enables well-being in older age” [3]. In Ghana, a national policy was developed in 2010 to provide a framework targeting key issues on ageing, titled the ‘National Ageing Policy: Ageing with Security and Dignity’, which acknowledged the need to address prevailing negative perceptions of ageing in society as well as economic challenges [4].

## 3 Finance

Retired persons who have worked in formal employment receive a pension from the Social Security and National Insurance Trust (SSNIT), a contributory statutory public pension trust. This however excludes a large proportion of older people as most would have worked in the informal sector [5]. The Livelihood Empowerment Against Poverty (LEAP) Programme is a state-funded initiative that targets the most vulnerable in society, including households with older persons living below the poverty line, by providing monthly monetary assistance [5]. LEAP is however limited in its actual reach and a large number of older adults remain financially constrained and depend on their children or younger relatives for support [6].

## 4 Healthcare

Older adults have a high prevalence of multimorbidity, which can pose a significant financing challenge for many of them [7]. Ghana’s National Health Insurance Scheme (NHIS), established in 2007 to replace the cash-and-carry system, exempts individuals aged 70 years and older (or 60 if they were SSNIT contributors) from subscription fees [6,8]. This is a commendable initiative that increases utilisation of formal healthcare services among older people; yet, there is room for improvement in actively promoting enrollment among older individuals, particularly in rural and peri-urban areas [9,10]. In addition, broadening the range of funded services to include more age-related conditions is necessary for ensuring comprehensive healthcare support for older people.

The World Health Organisation (WHO) recognises primary health services that are comprehensive and responsive to older people as a key part of age-friendly communities [3]. Ghana has a robust and well-organised primary care structure, with geriatrics being a growing yet recognised component of the Family Health Division of the Ghana Health Service [11]. Primary care facilities are the commonest contact points for most older persons seeking healthcare in Ghana [12]. Yet, evidence suggests that this system could be improved by providing further geriatric and gerontological education and training for health workers and students at all levels [13].

## 5 Education and research

As part of efforts to prepare the health service for an ageing population, a Fellowship in Geriatric Medicine was recently initiated by the Ghana College of Physicians and Surgeons and is one of the few of such programmes in Africa [14]. Through this initiative, geriatric clinics have been established in the major hospitals in the country, as well as in some of the community hospitals.

In 2016, the Center for Ageing Studies was established at the University of Ghana to advance multidisciplinary research and innovation in ageing in a local context [15].

Ghana is also one of the six sites for the WHO’s longitudinal Study on Global Ageing – SAGE (Waves 0, 1, 2 and 3) [16]. Using a nationally representative sample of older adults, the study explores the socio-demographics and health characteristics of this population [7].

## 6 Care facilities

Family caregiving has been the preferred and predominant modality for older people, but additional health and social care requirements exist in Ghana. Care homes tend to be unpopular in African societies as they are seen to contribute to a perception of family abandonment [17,18]. However, in certain situations, they may be necessary in providing skilled care and reducing loneliness and potential abuse in vulnerable persons. There are currently only a handful of care homes and daycare centers, clustered in the larger cities in Ghana. They are privately run and, hence, are often unaffordable for the average Ghanaian family. There is a need for regulation and development of these services, and to integrate them into the public health and social care system [6,12,17].

## 7 Dementia

WHO has recommended seven action areas for member states in response to dementia [19]. While some countries have made great strides in implementing them, progress has been relatively modest in Ghana. Dementia-friendly communities remain a much-needed component for advancing the conversation on Healthy Ageing, particularly in Sub-Saharan Africa, where the prevalence of dementia is rising sharply and outpacing the development of strategies and interventions [1].

## 8 Awareness and advocacy

Awareness of dementia in Ghana is generally low, with strong cultural and spiritual undertones as well as a low number of experts in this area influencing the diagnostic and care pathways for affected persons and their families [20]. This underscores the need for awareness programmes to dispel these notions, reduce stigma and maltreatment, and promote community support for people living with dementia [20,21].

In Ghana, advocacy has largely come from non-profit organisations such as Alzheimer’s Ghana, which was founded in 2012. This organisation has been involved in community education and advocacy, and in providing caregiver support and resources [[22], [23], [24]].

## 9 Research and policy

Ghana has initiated the drafting of a National Dementia Strategy, which is currently awaiting adoption and implementation [23,25]. Research into dementia in Ghana needs to be intensified and involve people living with dementia and their carers or families, in order to improve locally relevant dementia diagnostic and care pathways [22].

## 10 Caregiver support

In Ghana, caregiving is largely informal and carried out by families, particularly younger women, with limited education on the condition. This has significant physical, psychosocial and economic impacts. There is a need for post-diagnostic support for families and caregivers in this regard [26]. Alzheimer’s Ghana currently runs a virtual support group for caregivers to meet this need, but there is still room to integrate caregiver training, education and support into the mainstream primary or community health service.

## 11 Conclusion

This mini-review, which could not be exhaustive, demonstrates that Ghana has made notable progress in age-friendly initiatives, and these efforts have garnered attention from researchers, policymakers, and multidisciplinary professionals [27]. However, further efforts and support are needed in many areas, including the implementation of the National Ageing Policy, the adoption of a National Dementia Plan, intensifying dementia awareness campaigns to dispel stigma, improving financial support for older persons, and promoting education and research in geriatrics. In that, we advocate for a fully interdisciplinary and inter-agency approach that considers the unique cultural and lived perspectives of the Ghanaian society.

## Conflict of interests

The authors declare that they have no known competing financial interests or personal relationships that could have appeared to influence the work reported in this paper.
